# 101 Adrenergic Receptor Expression Is Increased in Carotid Smooth Muscle from Severely Burned Rats

**DOI:** 10.1093/jbcr/irac012.104

**Published:** 2022-03-23

**Authors:** Juquan Song, Jayson W Jay, Ye Wang, Ryan M Huebinger, Steven E Wolf

**Affiliations:** University of Texas Medical Branch, Galveston, Texas; University of Texas Medical Branch at Galveston, Galveston, Texas; University of Texas Medical Branch at Galveston, Galveston, Texas; UT Southwestern Medical Center, Dallas, Texas; University of Texas Medical Branch at Galveston, Galveston, Texas

## Abstract

**Introduction:**

Severe burn disrupts cardiovascular function which can lead to critical shock. To counteract cardiovascular collapse, there is a systemic increase of catecholamines released in response to severe burn. Previous studies showed that β1- adrenergic receptor (AR)_ protein expression was significantly increased in the cardiac right ventricles (RV) following burn injury, which is correlated with compromised cardiac dysfunction. Vascular smooth muscle contraction served to modulate blood pressure and improve circulatory perfusion. We hypothesize that ARs expression in major arteries are modified to initiate vascular functional changes following severe burn. In the current study, we report temporal ARs expression in murine carotid artery smooth muscle following severe burn.

**Methods:**

Thirty-four adult Sprague-Dawley male rats received a 40% total body surface area (TBSA) scald burn followed by fluid resuscitation using the Parkland formula. Control animals received a sham burn procedure. Animals were serially euthanized between 6 hours and 14 days after burn and endothelium-intact common carotid arteries were harvested for histological analysis.

**Results:**

Immunohistochemical staining data demonstrated expression of adrenergic receptors (AR) (α1, α2, β1, and β2) were differentially changed in response to injury over time. α1a-AR expression significantly increased within the carotid artery tunica media 7-days after burn (p< 0.05). As a negative feedback of inhibitory of norepinephrine signaling, AR-α2a expression did not significantly change. AR-β1 expression also had no change over time after burn. Interestingly, functioning to relax vascular smooth muscle, a significant elevation of β2-AR expression within the carotid artery tunica media was observed only at 1-day after burn (p< 0.05).

**Conclusions:**

In summary, immunohistochemistry showed that carotid arterial adrenergic receptor expressions of α1a-AR and β2-AR are significantly altered in response to severe burn, which may contribute to vascular contractility in burn rats.

